# Using Bayesian dynamical systems, model averaging and neural networks to determine interactions between socio-economic indicators

**DOI:** 10.1371/journal.pone.0196355

**Published:** 2018-05-09

**Authors:** Björn R. H. Blomqvist, Richard P. Mann, David J. T. Sumpter

**Affiliations:** 1 Uppsala University, Department of Mathematics, Uppsala, Sweden; 2 University of Leeds, School of Mathematics, Leeds, United Kingdom; Arizona State University & Santa Fe Institute, UNITED STATES

## Abstract

Social and economic systems produce complex and nonlinear relationships in the indicator variables that describe them. We present a Bayesian methodology to analyze the dynamical relationships between indicator variables by identifying the nonlinear functions that best describe their interactions. We search for the ‘best’ explicit functions by fitting data using Bayesian linear regression on a vast number of models and then comparing their Bayes factors. The model with the highest Bayes factor, having the best trade-off between explanatory power and interpretability, is chosen as the ‘best’ model. To be able to compare a vast number of models, we use conjugate priors, resulting in fast computation times. We check the robustness of our approach by comparison with more prediction oriented approaches such as model averaging and neural networks. Our modelling approach is illustrated using the classical example of how democracy and economic growth relate to each other. We find that the best dynamical model for democracy suggests that long term democratic increase is only possible if the economic situation gets better. No robust model explaining economic development using these two variables was found.

## 1 Introduction

In recent years, an extensive amount of data describing the state of social and economic systems has become available. For example, the World Bank collects statistics on global development data since 1960, and has made them freely available in the form of indicator variables of education, health, income, but also pollution, science and technology, government and policy performances [1]. Data availability has opened up possibilities for a vast number of studies on evolution of the political, economical and sociological aspects of global development. Some examples include: causes of economic growth [2]; impact of democracy on health, schooling and development [3, 4]; globalization and changes in societal values [5]; and relationships between liberalism, post-materialism and freedom [6]. Studies of social systems often consider different scales—e.g. community, municipality, states, and countries,— but address a common fundamental question: is it possible to extract the underlying essential relationships and development patterns of indicator variables from time series data [7]? Knowing such relationships would constitute a significative step towards interpreting, predicting and possibly controlling, social and economical development.

Linear and non-linear interactions between indicator variables are common in social systems [8–10], but time series data are often noisy and incomplete, posing significant challenges in the identification of such fundamental relationships.

**Fig 1:**
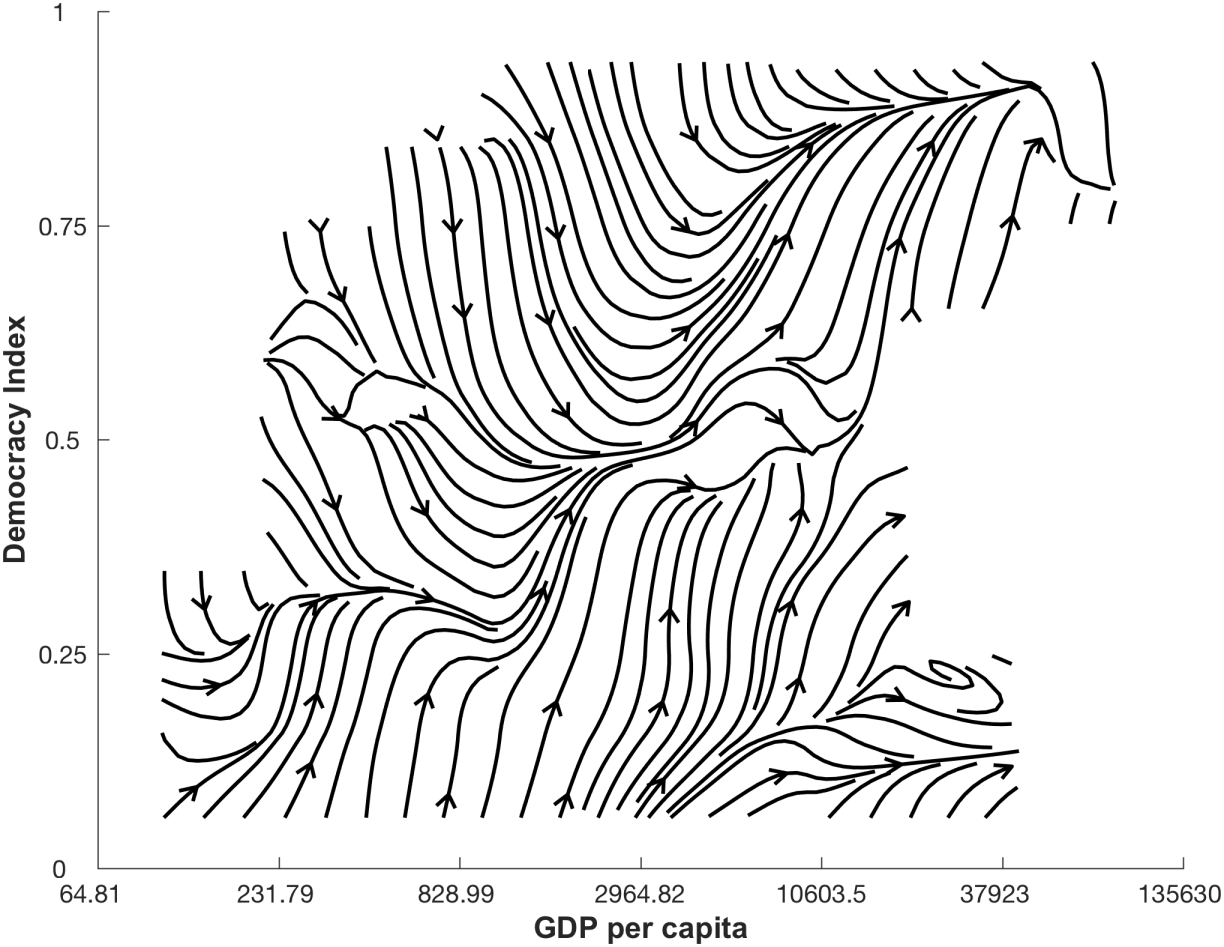
Naive approximation of the non-linear dynamics relationship between democracy (*D*) and log GDP per capita (*G*). The average change of all data points in the *G* and *D* directions is calculated in the state space of 100 equally sized regions visualized using an interpolating stream-slice plot. Where there are no lines, there is no data available.

Let us take as an example the extensively studied, and hotly debated, relationship between democracy (*D*) and economic development (*G*) measured as GDP per capita [11–15]. In our study, time series data for *D* is based on the Freedom House political rights and civil liberties scores [16–18], weighted by the human-rights-performance, taking values between zero and one. The World Bank provides time series data of *G* in U.S. dollar and in total we include data for 174 countries from 1981 and 2006, for a total of 3445 data points, averaging 19 data points per country. The dynamic relationship underlying these data can be conveniently represented as a vector field in the (*D*, *G*) state space, as shown in Fig 1. We obtain this visual representation by computing the change of all data points in the *G* and *D* directions, we then divide the state space into 100 equally sized regions (10 by 10), average the changes in the data points within each area, and finally visualize the resulting vectors using an interpolating stream-slice plot. Although this is a naive approximation, Fig 1 provides a picture of the non-linear nature of the democracy-GDP relationship.

It still remains unclear how much of the observed pattern in Fig 1 is due to a genuine relationship between the indicators and how much is random noise in data. Such aspect as non-linearity and noise in the data significantly lowers the accuracy of the equation-based statistical models that one would traditionally use to fit data.

Within the fast-growing field of machine learning, artificial neural networks (ANN) are a simple, useful and accurate tool for modeling non-linear and complex systems, even when the available data is noisy [19–21]. Based on nonparametric estimations, this method can serve as a universal approximator [22], enabling fitting of data without constraints and guidance from theory, and is widely used in forecasting, modeling and classification. Since the 1990s, neural networks have been applied in fields as diverse as medical diagnosis [23], forecasting groundwater levels [24], speech recognition [25, 26], and species determination in biology [27]. Recently, the nonlinearities characterizing social-economical systems have lead researchers in this area to turn to machine learning techniques [28]. Models obtained with ANN and similar prediction-oriented methods accurately reproduce empirical patterns. However results from ANNs essentially remain black boxes [29], making it difficult to translate from a fitted model to insights into the relationships between indicators.

Recently, Ranganthan et al. [15] introduced an approach to analyze time series data of social indicators that starts to bridge the gap between black-box machine learning algorithms and traditional statistical models by finding coupling functions [30] of the dynamical socio-economics interactions. Coupling functions are used for studying dynamics in many applications, such as: chemistry [31–33]; cardiorespiratory physiology [34, 35]; neural science [36]; communications [37]; and social science [3, 15, 38–41]. Ranganthan et al. [15] developed a Bayesian algorithm to trade-off between high explanatory power and complexity when selecting the best polynomial model to fit data. With this approach they were able to identify non-linear, dynamical relationships between indicator variables. In particular, when studying the relationship between democracy and economic development they found the best function to describe changes in democracy to be

$$\begin{array}{c} \frac{dD}{dt}=0.11G^{3}-0.067\frac{D}{G}. \end{array}$$ (1)

According to this expression, democracy increases once GDP per capita has reached a certain threshold that depends on the democracy level itself. The best model for GDP per capita was

$$\begin{array}{c} \frac{dG}{dt}=0.014+0.0064DG-0.02G, \end{array}$$ (2)

telling us that most of the change in GDP would be explained by a positive constant which is decreased in richer but less democratic countries. Their approach has been extended to problems with more than two variables, and used to analyze human development [15, 42, 43], the environment [44], democracy [3] and school segregation [40].

The aim of this paper is twofold. On the one hand, we improve on the approach of Ranganthan et al. [15] to fit equation-based ‘best models’, through Bayesian linear regression and now on all tested possible model combinations. In particular, by adopting a mathematical convenient and practical class of priors, we are able to get closed form expressions for the the marginal likelihood of each model, to accurately compare a large number of models (while in [15] this number was limited to one model per number of terms in model), and to significantly speed up computational time. Furthermore, the novel aspect of assessing all potential models allows us to rank them and to discuss the relative importance and robustness of different linear and non-linear terms and their combinations by studying how often they recur. On the other hand, we compare our improved approach, i.e. the (1) Bayesian-selected ‘best model’, with two other approaches for modelling time series in social economical systems, i.e. (2) model averaging (over a subset of models obtained with our Bayesian approach) and (3) artificial neural networks. Our ultimate aim is to select the best models distinguishing genuine relationships between indicator variables from random noise, retaining prediction estimates and in the meantime the highest explanatory power.

The paper is structured as follows. In the methods section (2) we describe the general framework we use to represent time series data (2.1), and the three approaches we consider to fit these data: our improved Bayesian-selected best model (2.2), Bayesian model averaging (2.3) and neural networks (2.4). In section 3 we report the results obtained by applying these three approaches on a case study, the relationship between democracy and GDP per capita. In section 4 we compare our Bayesian best model approach to the other two, discuss pros and cons, and compare our results on democracy and GDP with other studies.

## 2 Methods

### 2.1 Representation of time series data

We assume the social systems we investigate are described by *n* indicator variables, as democracy and GDP per capita in the example above (where *n* = 2). Each individual entity *m* in this system, e.g. a country, a state, a city, provides a discrete time series for each indicator variable *x*_*i*_(*t*), *i* ∈ [1, 2, …, *n*] during a time period *T*. Here, we interpret these individual time series as realizations of paths of the same global system, but starting from different initial conditions. In other words, by this we mean that we assume that all entities within the investigated social system is governed by the same dynamical relations between indicator variables and their individual time series are stochastically realizations of the dynamics staring from different initial conditions. This corresponds with discarding the individual, possibly large, differences between entities, assuming their evolution is affected only by their position in the indicators state space (*x*_1_, *x*_2_, …, *x*_*n*_). These assumptions enable us to fit the individual time series to obtain a global model for the dynamical changes in the indicator variables. In particular, we aim at giving an accurate estimate of global indicators’ changes between time *t* and *t* + 1 depending only on their value at time *t*, i.e. on their position in the state space.

### 2.2 Bayesian best model

The Bayesian ‘best model’ selection we propose here fits time series data for the indicator variables to a model constituted by a system of *n* ordinary differential equations

$$\begin{array}{ccc} \frac{dx_{1}}{dt} & = & f_{1}\left(x_{1},x_{2},...,x_{n}\right)+\epsilon_{1}\\ \frac{dx_{2}}{dt} & = & f_{2}\left(x_{1},x_{2},...,x_{n}\right)+\epsilon_{2}\\ & \vdots & \\ \frac{dx_{n}}{dt} & = & f_{n}\left(x_{1},x_{2},...,x_{n}\right)+\epsilon_{n}. \end{array}$$ (3)

Here, *f*_1_, …, *f*_*n*_ are unknown coupling functions of the indicator variables and we assume uncorrelated random noise terms *ϵ*_*i*_. The selection process takes the three following steps: (1) Define all the possible model configurations; (2) fit the data to these configurations through Bayesian regression; and (3) compare model configurations and choose the best suitable model. Notice that although for notation convenience we write these equations in continuous time, we actually fit difference equations as available data is often reported at discrete times.

#### Step 1: Possible model configurations

To enhance interpretability, we choose to approximate the functions *f*_*i*_ with polynomials consisting of linear and non-linear combinations of the indicator variables. Typically, we use terms up to order three and define a model configuration $M_{i}$ as any subset of the coefficients of such combination. Including a considerable amount of non-linear terms allows for multi-stable states which are frequently found in social systems [15, 45]. For example, in a model with *n* = 2 our preliminary choice of functions is:

$$\begin{array}{ccc} f(x_{1},x_{2}) & = & a_{0}+\frac{a_{1}}{1+x_{1}}+\frac{a_{2}}{1+x_{2}}+a_{3}x_{1}+a_{4}x_{2}\\ & & +\frac{a_{5}}{\left(1+x_{1}\right)\left(1+x_{2}\right)}+a_{6}\frac{x_{1}}{1+x_{2}}+a_{7}\frac{x_{2}}{1+x_{1}}\\ & & +a_{8}x_{1}x_{2}+a_{9}x_{1}^{2}+a_{10}x_{2}^{2}+\frac{a_{11}}{{\left(1+x_{1}\right)}^{2}}\\ & & +\frac{a_{12}}{{\left(1+x_{2}\right)}^{2}}+a_{13}x_{1}^{3}+a_{14}x_{2}^{3}+\frac{a_{15}}{{\left(1+x_{1}\right)}^{3}}\\ & & +\frac{a_{16}}{{\left(1+x_{2}\right)}^{3},} \end{array}$$ (4)

and a model configuration $M_{i}$ would be any subset of the coefficients {*a*_0_, …, *a*_16_} for a total of 2^17^ = 131,072 configurations. This choice follows [15], but we have rescaled the variables to take values between zero and one and included a +1 in terms with denominators to avoid singularities. The chosen functional form of *f*_*i*_ offers the highest degree of flexibility for systems with relatively small *n*, but it may be adjusted by adding or removing terms. We have tested our Bayesian framework on normalized input variables, in a setup without variables in denominators. The resulting ‘best models’ provided similar dynamics, but we argue our proposed model configurations are better for interpretation.

#### Step 2: Fit data to model configurations

In this step we obtain the coefficient values by applying Bayesian linear regression [46, 47] on all the possible model configurations. The Bayesian linear regression practically consists in (1) assigning prior distributions to the unknown coefficients in each configuration; (2) Get the likelihood of the coefficients given the data; (3) Determine the posterior distribution of the coefficients by combining the priors and the likelihood using Bayes theorem [47].

In standard linear regression, one fits *n* response variables **y** = *x*(*t* + 1) − *x*(*t*) to the explanatory variables **X**. The explanatory variables *X* is a *n* × *p* design matrix consisting of linear and nonlinear terms in the tested model configuration $M_{i}$, where *n* is the number of observations and p is the number of terms in the tested model configuration. The model for the response variable is typically divided into two components, deterministic and gaussian noise:

$$\begin{array}{c} y=X\beta +\epsilon \end{array}$$ (5)

where *β* is a *p* × 1 vector of slope coefficients and *ϵ* is a *n* × 1 vector of gaussian noise. For the different model configurations $M_{i}$ (Eq 4) we consider *p* ∈ [1, …, 17], being the number of terms in the investigated model. For example, the one model where all terms is included, we have *β* = (*a*_0_, *a*_1_, …, *a*_16_)^⊤^ and *p* = 17 (Eq 4).

A common way of finding an approximation of the unknown slope coefficients $\beta \in R^{p}$ is finding maximum likelihood estimates ${\hat{\beta }}_{MLE}$ through [47]:

$$\begin{array}{c} {\hat{\beta }}_{MLE}={\left(X^{T}X\right)}^{-1}X^{T}y. \end{array}$$ (6)

In principle, by evaluating the log-likelihood, i.e. the logarithm of the probability of observing the data given model parameters of model $M_{i}$, we could find the model that best represents data. The likelihood in our setting is [46]

$$\begin{array}{c} P\left(y|X,\beta ,\sigma^{2}\right)=N(X\beta ,\sigma^{2}I) \end{array}$$ (7)

where *σ*^2^ is the regression variance. However, by definition the likelihood increases with the number of terms in the model, which would give us overcomplicated and difficult to interpret equations.

Our approach faces this problem by using a Bayesian approach and assigning prior distributions *p*(*β*, *σ*^2^) on the coefficients *β* and *σ*^2^. The priors are assigned only on the coefficients of the assumed prior model configurations, later after all of the data is presented, the model coefficients are updated. Since we introduce all of the available data at the same time, the priors are used once in our modelling approach and are assumed to be the same for all the different entities (countries in our case) since they are assumed to be different realisations of the same social system. Combining prior knowledge and the likelihood of the data using Bayes theorem gives us the posterior distribution of coefficients [46]:

$$\begin{array}{c} p\left(\beta ,\sigma^{2}|y,X\right)=\frac{P\left(y|X,\beta ,\sigma^{2}\right)p\left(\beta ,\sigma^{2}\right)}{p(y|X)}. \end{array}$$ (8)

A flat prior distribution *p*(*β*, *σ*^2^) would give us the maximum likelihood estimate (6), assuming that the MLE lies within the range of the prior. This approach was for example used in [15]: in their implementation, they first found the model configurations with the highest log likelihood and then numerically calculated the marginal likelihood using Monte Carlo techniques for those models.

Here, we use a Normal Inverse Gamma (NIG) distributed prior with parameters (**m**_0_,**V**_0_, *a*_0_, *b*_0_):

$$\begin{array}{ccc} p(\beta ,\sigma^{2}) & = & NIG(m_{0},V_{0},a_{0},b_{0})\\ & = & \frac{b_{0}^{a_{0}}\sigma^{-2(a+(k/2)+1)}}{{\left(2\pi \right)}^{k/2}{\left|V_{0}\right|}^{1/2}\Gamma \left(a_{0}\right)}\times \text{exp}\left(\frac{2b_{0}-{\left(\beta -m_{0}\right)}^{\prime }V_{0}^{-1}\left(\beta -m_{0}\right)}{2\sigma^{2}}\right) \end{array}$$ (9)

This choice has the double advantage of adjusting the punishment of overcomplicated models (more about this later) and, since it is a conjugate prior, of allowing for closed form calculations. Indeed, combining the likelihood with the NIG prior gives a NIG posterior distribution with updated parameters (**m**_∗_, **V**_∗_, *a*_∗_, *b*_∗_) [46],

$$\begin{array}{ccc} m_{*} & = & {\left(V_{0}+X^{T}X\right)}^{-1}\left(V_{0}m_{0}+X^{T}y\right)\\ V_{*} & = & V_{0}+X^{T}X\\ a_{*} & = & a_{0}+n/2\\ b_{*} & = & b_{0}+\frac{1}{2}\left(m_{0}^{T}V_{0}m_{0}+y^{T}y-m_{*}^{T}V_{*}m_{*}\right). \end{array}$$ (10)

The best coefficients *β* and *σ*^2^ would then be given by the posterior mean, $\hat{\beta }=m_{*}$ respectively $\sigma^{2}=\frac{b_{*}}{a_{*}-1}$ for a_*_ > 0.

A similar but simpler choice for the prior which is commonly used is the Zellner g-prior [48], specified by

$$\begin{array}{ccc} m_{0} & = & 0\\ V_{0} & = & \frac{1}{g}\left(X^{T}X\right)\\ a_{0} & \to & 0\\ b_{0} & \to & 0 \end{array}$$ (11)

This prior features convenient choices of the hyper-parameters, hence utilizing fewer parameters by letting *a* and *b* going to zero, but retains the same essential features of the NIG prior. The parameters are set to be very small, but can’t be set to zero because this would brake down Eq (15).

We choose the data dependent unit information prior *g* = "number of data points" [49], which effectively provides the same amount of information as one observation: the ${\hat{\beta }}_{MLE}$ has precision (**X**^*T*^ **X**)^−1^/*σ*^2^ and can be interpreted as the amount of information contained in *n* observations. The unit information prior is then (**X**^*T*^ **X**)^−1^/(*nσ*^2^), i.e. “one-*n*^*th*^” of the precision [50]. By using the same *g*-prior for all model configurations $M_{i}$ we therefore punish all overcomplicated configurations in the same way. Moreover, this choice of *g* also puts more weight on the data and less on the prior when there is a lot of data available.

Another possible assumption on the prior distribution is to put the covariances of the explanation variables to zero,

$$\begin{array}{ccc} m_{0} & = & 0\\ V_{0} & = & diag(\frac{1}{g}X^{T}X)\cdot I\\ a_{0} & \to & 0\\ b_{0} & \to & 0 \end{array}$$ (12)

where **I** is the identity matrix. This makes the prior behave like in ridge regression, by adding small values, inversely proportional to the variance of each explanation variable, on the diagonal entities of *X*^*T*^ *X*. This choice of prior penalizes the least efficient parameters i.e. explanation variables with the most variance the most, and overcome ill-conditioned problems by punishing model configurations with collinearities. This assumption is motivated since we potentially use highly collinear explanation variables in some of the model configurations e.g. $\frac{1}{\left(1+x_{1}\right)}+\frac{1}{{\left(1+x_{1}\right)}^{2}}+\frac{1}{{\left(1+x_{1}\right)}^{3}}$ which can cause highly unstable estimations $\hat{\beta }$ [51]. Since we are looking for models with high explanatory power, collinear terms are especially unwanted, since they do not add to the understanding i.e. we want simple models without two terms describing similar behavior.

We tested both the standard g-prior (Eq 11) and an updated g prior (Eq 12) on our example with democracy and log GDP per capita. Models with low number of terms got the same best model configurations both for democracy and log GDP per capita, but using the standard g-prior, collinear terms dominated the models using more terms, especially for log GDP per capita. Using Eq (4) as our preliminary choice of functions, with many possible collinearities, thereby leads up to choose g (Eq 12).

The posterior mean of the coefficient *β* then becomes

$$\begin{array}{c} \hat{\beta }=(X^{T}X+diag{\left(\frac{1}{g}X^{T}X)\cdot I\right)}^{-1}\left(X^{T}y\right). \end{array}$$ (13)

Notice that as *g* → ∞, $\hat{\beta }$ tends to the maximum log likelihood estimate. Conversely, *g* → 0 would force the posterior towards the prior distribution making the inference impossible.

#### Step 3: Comparing model configurations

Once we fit each model configuration $M_{i}$ to the same dataset by using the same *g*-prior, we compare them by using their marginal likelihood to punish over complicated models, i.e those with many terms, by taking account for the uncertainty in the model parameters. The marginal likelihood $p(y,X|M_{i})$, is a measure of the probability of observing the data under the hypothesis that the model configuration $M_{i}$ is true. This probability, also referred to as the model evidence [52], is calculated by integrating over the parameters in the model:

$$\begin{array}{c} p\left(y,X|M_{i}\right)=\int \int p\left(y,X|\beta_{i},\sigma^{2}\right)p\left(\beta ,\sigma^{2}\right)d\beta_{i}d\sigma^{2} \end{array}$$ (14)

In our conjugate setting this integral can be computed analytically and the marginal likelihood for $M_{i}$ is [46]:

$$\begin{array}{c} p\left(y,X|M_{i}\right)=\frac{1}{{\left(2\pi \right)}^{n/2}}\sqrt{\frac{\left|V_{0}^{i}\right|}{\left|V_{*}^{i}\right|}}\cdot \frac{b_{0}^{a_{0}}}{{b_{*}}^{a_{*}}}\cdot \frac{\Gamma (a_{*})}{\Gamma (a_{0})}. \end{array}$$ (15)

The intuition behind how the marginal likelihood punishes over-complicated models is the following; when the model complexity goes up, we spread out the prior over more terms and thereby have to perform integration over both ‘good’ and ‘bad’ terms, resulting in lower prior mass on the ‘good’ terms resulting in a lowered marginal likelihood. The marginal likelihood is also affected by our *g* parameter.

To compare two configurations $M_{i}$ and $M_{j}$ we use the Bayes factor. The Bayes factor is the posterior odds divided by the prior odds, which is equal to the quotent of the marginal likelihoods (Eq 15):

$$\begin{array}{c} BF\left(M_{i},M_{j}\right)=\frac{{\left|V_{0}^{j}\right|}^{1/2}{\left|V_{*}^{i}\right|}^{1/2}{\left(b_{*}^{j}\right)}^{a_{*}}}{{\left|V_{0}^{i}\right|}^{1/2}{\left|V_{*}^{j}\right|}^{1/2}{\left(b_{*}^{i}\right)}^{a_{*}}}. \end{array}$$ (16)

The higher the Bayes factor, the better the model $M_{i}$ is compared to $M_{j}$. In our study we compare all model configurations to the constant change model $M_{\text{const}}$ i.e. constant change between times *t* and *t* + 1. By comparing all models to this benchmark model we can rank all possible models.

Additionally, we perform a visual comparison by plotting the dynamical changes in the phase space as described by each configuration, and compare the coefficient of determination (*R*^2^) of different model configurations. The *R*^2^ value gives us the proportion of the total variation in the data picked up by our models. The *R*^2^ value is computed by

$$\begin{array}{c} R^{2}=1-\frac{\sum_{i}{\left(f_{i}-\bar{y}\right)}^{2}}{\sum_{i}{\left(y_{i}-\bar{y}\right)}^{2}}. \end{array}$$ (17)

where $\bar{y}$ is the mean change and *y*_*i*_ is data points. Therefore a higher *R*^2^ value corresponds to a higher explanatory power of the given configuration.

### 2.3 Bayesian model averaging

Bayesian model averaging weights the obtained model configurations by their marginal likelihood and combines them into an ‘average’ model [53, 54]. This process integrates information from different models, providing a way of handling uncertainty and reducing the risk of overestimation [53, 55–57]. In this way, the uncertainty in model selection is treated in the same way as parameter uncertainty within a single model. In what follows we will compare the performances of three Bayesian average models obtained by combining the 1%, 10%, and 50% of the highest marginal likelihood configurations obtained with the process described in section 2.2.

### 2.4 Artificial neural network

**Fig 2:**
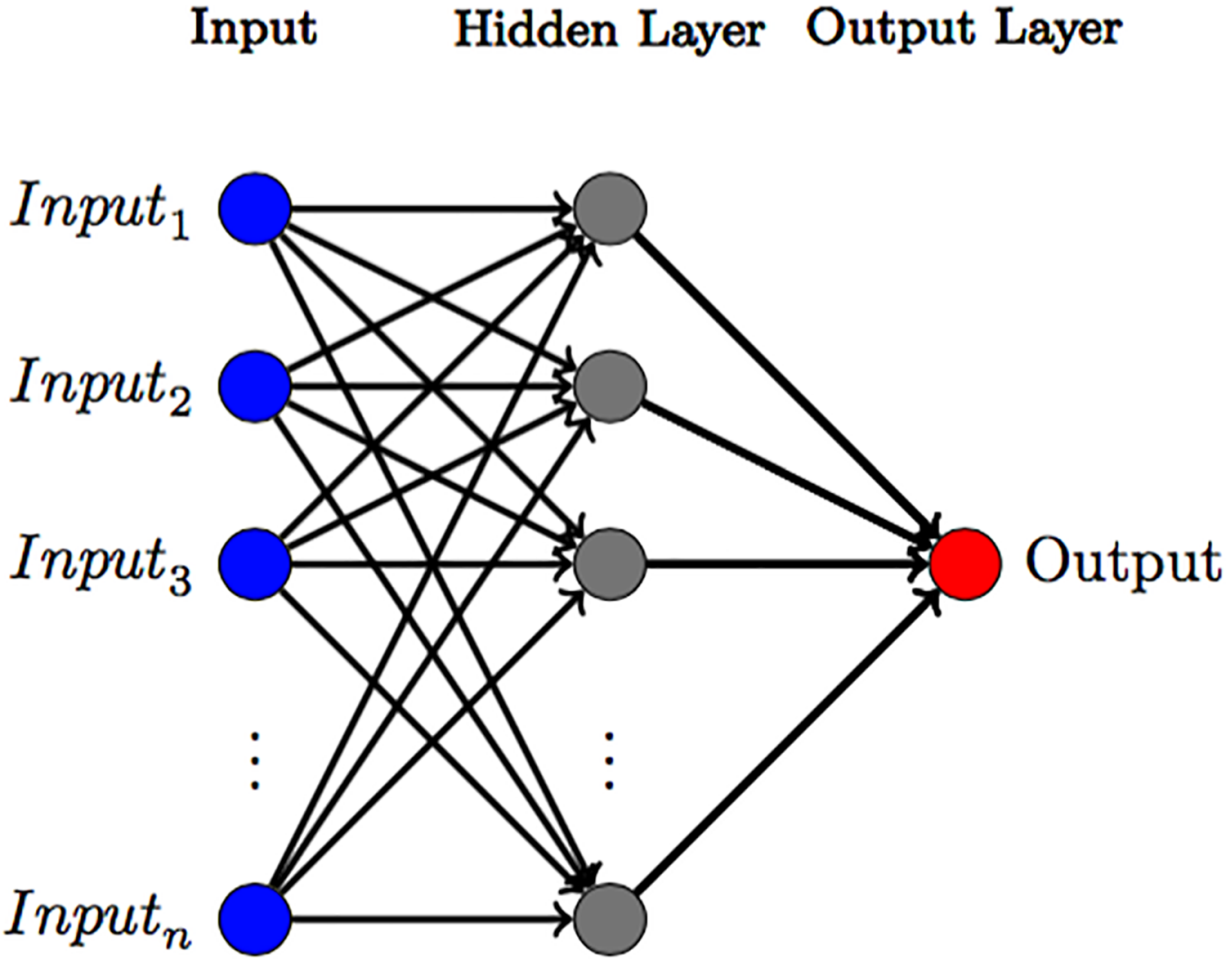
Diagram of an feed-forward neural network with one hidden layer. This figure shows a generic feed forward neural network with one hidden layer. The neural network uses *n* input variables and produce one output variable after passing through the network.

We use the Matlab neural network package *fitnet* [58] to get a nonparametric estimate of the dynamical evolution of indicators that we can use as a benchmark to compare our Bayesian approach. *fitnet* is a feedforward neural network using a tan-sigmoid transfer function and a linear transfer function in the output layer [58]. In this paper, we choose to use one single hidden layer and to vary the number of neurons to adjust for the level of fit of the network (see Fig 2).

In order to find a suitable number of neurons, not underestimating nor overestimating the network, we perform K-fold cross validation [59]. We use five folds and find the mean *R*^2^ values for 1000 neural networks using 1-10 neurons (for each of the five folds). It is worth pointing out that since we assumed Gaussian noise above, the *R*^2^ is related directly to likelihood of the model. The neural network model with the best cross-validated number of neurons (highest *R*^2^) is called the ‘best neuron network model’. After a suitable number of neurons is chosen, we train the neural networks using 70% of the available data, and then validating and testing the model using 15% respectively. We compare our ‘best’ neural network model with two additional neural networks, namely one neural network model using only one neuron, representing an underestimating model and a model using ten neurons representing an overestimated model.

### 2.5 Surrogate data testing

To test the validity of the coupling functions we used surrogate data testing [60–62]. We generate surrogate data using the best model configurations of each coupling function and use bootstrapped initial data from the original dataset. The validity of the model from the original data is strengthen if we can reproduce the models generated from the surrogate data and thereby provide evidence that it was not just created by chance.

Specifically, the initial surrogate data is generated for 248 (number of countries and other sub-regions in the original data sets, including those regions without any data) countries using random sampling from our original data with replacement. We then apply the coupling functions i.e. best explicit functions, with corresponding noise terms, to simulate the changes the investigated indicator variables, producing data for an additional data 25 time-steps.

## 3 Results: Democracy vs. log GDP per capita

We now apply the three approaches to the same case study: the relationship between democracy (*D*) and log GDP per capita (*G*). Formally, the relationship between *D* and *G* takes the form of two coupled differential equations:

$$\begin{array}{ccc} \frac{dD}{dt} & = & f_{D}\left(D,G\right)+\epsilon_{D}\\ \frac{dG}{dt} & = & f_{G}\left(D,G\right)+\epsilon_{G} \end{array}$$ (18)

Firstly, we are interested in testing each approach for extracting the dynamical features of the coupled change in Democracy and log GDP per capita, i.e. the best fit of *f*_*D*_ and *f*_*G*_ to the time series data. We focus on the selection of the best functional form for *f*_*D*_ and *f*_*G*_ through our Bayesian best model approach. Secondly, we cross-compare the performances of the three approaches and we analyze the recurrence of single and combined terms in the functions *f*_*D*_ and *f*_*G*_ extracted by the Bayesian best model. This allows us to assess the robustness of our approach and to see to what extent it trades-off between accuracy and interpretability.

### 3.1 Best fit Bayesian models

We start from the general *n* = 2 model described by Eq (4). The model configurations are defined by subsets of the coefficients [*a*_0_, …, *a*_16_]. All possible combinations of these coefficients would give a total of 2^17^ model configurations. For simplicity and interpretability, we will restrict our analysis to model configurations with a maximum of 5 terms, for a total of ∑k=15(17k)=9,401 investigated configurations M.

The best 1 to 5 term models M for democracy *f*_*D*_(*D*, *G*) and log GDP per capita *f*_*G*_(*D*, *G*) extracted by our approach are shown in Table 1 and ranked according to the logarithm of the Bayes factor (Eq 16) with respect to a constant model $M_{\text{c}}$, and to the coefficient of determination *R*^2^ (Eq 17).

**Table 1:** Comparison of best models for democracy and log GDP per capita. The main three groups of rows correspond to the three tested approaches, each shaded sub-row corresponds to the best model for the corresponding approach. For the Bayesian best model, columns display (left to right): the top 1-5 terms models, their log Bayes factor(BF), their configuration ranking (out of 9401), and *R*^2^ value. We report the *R*^2^ values for the average models and feedforward Neural Network models.

	**Democracy**			
**Model**: *f*_*D*_(*D*, *G*)	$\text{log}(\text{BF}(M,M_{\text{const}}))$	Rank	*R*^2^	
0.013/(1 + *D*)^3^	12.6	8397	0.7%	
0.18*DG* − 0.15*D*^2^	47.4	251	3.0%	
0.16*DG* − 0.14*D*^2^ + 0.01/(1 + *D*)^3^	54.0	1	3.6%	
0.34*D* − 0.5*D*/(1 + *G*) + 0.03/(1 + *G*)^3^ − 0.09*D*^3^	52.9	4	3.9%	
0.2*DG* − 0.09*D*/(1 + *G*) − 0.05*G*^3^ + 0.02/(1 + *D*)^2^ − 0.1*D*^3^	50.3	41	4.0%	
Average model (1 procent)	-	-	3.8%	
Average model (10 procent)	-	-	3.8%	
Average model (50 procent)	-	-	3.5%	
Neural Network (1 Neuron)	-	-	3.6%	
Neural Network (4 Neurons)	-	-	4.1%	
Neural Network (10 Neurons)	-	-	4.7%	
	**log GDP per capita**			
**Model**: *f*_*G*_(*D*, *G*)	$\text{log}(\text{BF}(M,M_{\text{const}}))$	Rank	*R*^2^	
0.011	0.0	360	0.0%	
0.02*D* + 0.01/(1 + *D*)^3^	8.2	1	0.7%	
0.06*D*^2^ + 0.01/(1 + *D*)^3^ − 0.05*D*^3^	4.8	21	0.9%	
0.0005*G*^3^ + 0.06*D*^2^ + 0.01/(1 + *D*)^3^ − 0.05*D*^3^	0.6	248	0.9%	
0.35 + 0.01*D*/(1 + *G*) − 1.5/(1 + *G*)^2^ − 0.14*G*^2^ + 1.21/(1 + *G*)^3^	0.3	279	1.5%	
Average model (1 procent)	-	-	0.7%	
Average model (10 procent)	-	-	1.0%	
Average model (50 procent)	-	-	0.9%	
Neural Network (1 Neuron)	-	-	0.5%	
Neural Network (6 Neurons)	-	-	1.8%	
Neural Network (10 Neurons)	-	-	2.2%	

Except for the one-term model, all models for democracy include both democracy and log GDP per capita. The one-term model depends only on *D*, indicating that democracy typically grows with a rate that slows down as democracy itself increases. The best two-terms model can be rewritten in the form *D*(0.18*G* − 0.15*D*) suggesting a threshold at *D* = 1.2*G*. When *D* > 1.2*G* democracy decreases and when *D* < 1.2*G* democracy increases.

The best model for the change in democracy has three terms

$$\begin{array}{c} f_{D}\left(D,G\right)=0.16DG-0.14D^{2}+\frac{0.01}{{\left(1+D\right)}^{3}}, \end{array}$$ (19)

which is a combination of the one-terms and the two-terms models. In particular, the two first terms 0.16*DG* − 0.14*D*^2^ indicate the existence of a threshold at *D* = 1.14*G* as in the two-term model, but with updated coefficients. The four- and five-term models have a larger Bayes factor than the one- and two-term models, but are not as good as the three-term model, indicating that our approach successfully trades-off between accuracy and complexity in fitting this indicator.

For the log GDP per capita, the best model with only one term gives a constant rate of economic growth of 1.1% per year. Model configurations with two and three terms depend only on democracy, while models with four and five terms include both democracy and log GDP per capita. In terms of Bayes factor, the best model has two terms

$$\begin{array}{ccc} f_{G}\left(D,G\right) & = & 0.008D+\frac{0.005}{{\left(1+D\right)}^{3}}. \end{array}$$ (20)

This model suggests that a potential driver of change in log GDP per capita is democracy. The first term indicates that log GDP per capita increases when democracy increases. The second term is also positive but gives a bigger contribution when democracy levels are low. As a result, GDP grows slowest when *D* = 0.116, a level corresponding to rather undemocratic countries such as Burundi, Dominican Republic, Hungary in 1981, or Angola and Guinea in 2006. As *D* increases past this level the economy grows more rapidly.

### 3.2 Comparison of the three methods

Overall, our approach identifies two best models for democracy and GDP both featuring a relatively low amount of terms, which would make them easy to interpret. However, while the measures for the goodness of fit data are high in the case of democracy, this is not the case for GDP. This might indicate our modelling approach is not suitable for describing GDP data. Therefore, we first compare our best model to the fits given by the Bayesian average model and the neural network, and then investigate how often certain terms appear in the best 100 models extracted with our Bayesian approach.

The simplest way of comparing the three considered approaches is through the coefficient of determination *R*^2^ (see Table 1). Our Bayesian best model for democracy has a *R*^2^ of 3.6%, the best model obtained by model averaging is obtained by including the 10% top configurations and has a *R*^2^ of 3.8%. The best neural network model (four neurons) gives the best fit for the democracy dataset, with a *R*^2^ of 4.1%, but does not provide equations that we can easily interpret. Interestingly, the *R*^2^ value of our Bayesian best model is very close to the *R*^2^ of the best neural network, supporting the claim that our Bayesian best model is close to the best possible fit to the given data set, with the additional advantage that it provides an explicit form for *f*_*D*_. In the table we also include neural network models with one and ten neurons for comparison with to an under-, respectively over-trained neural networks.

**Fig 3:**
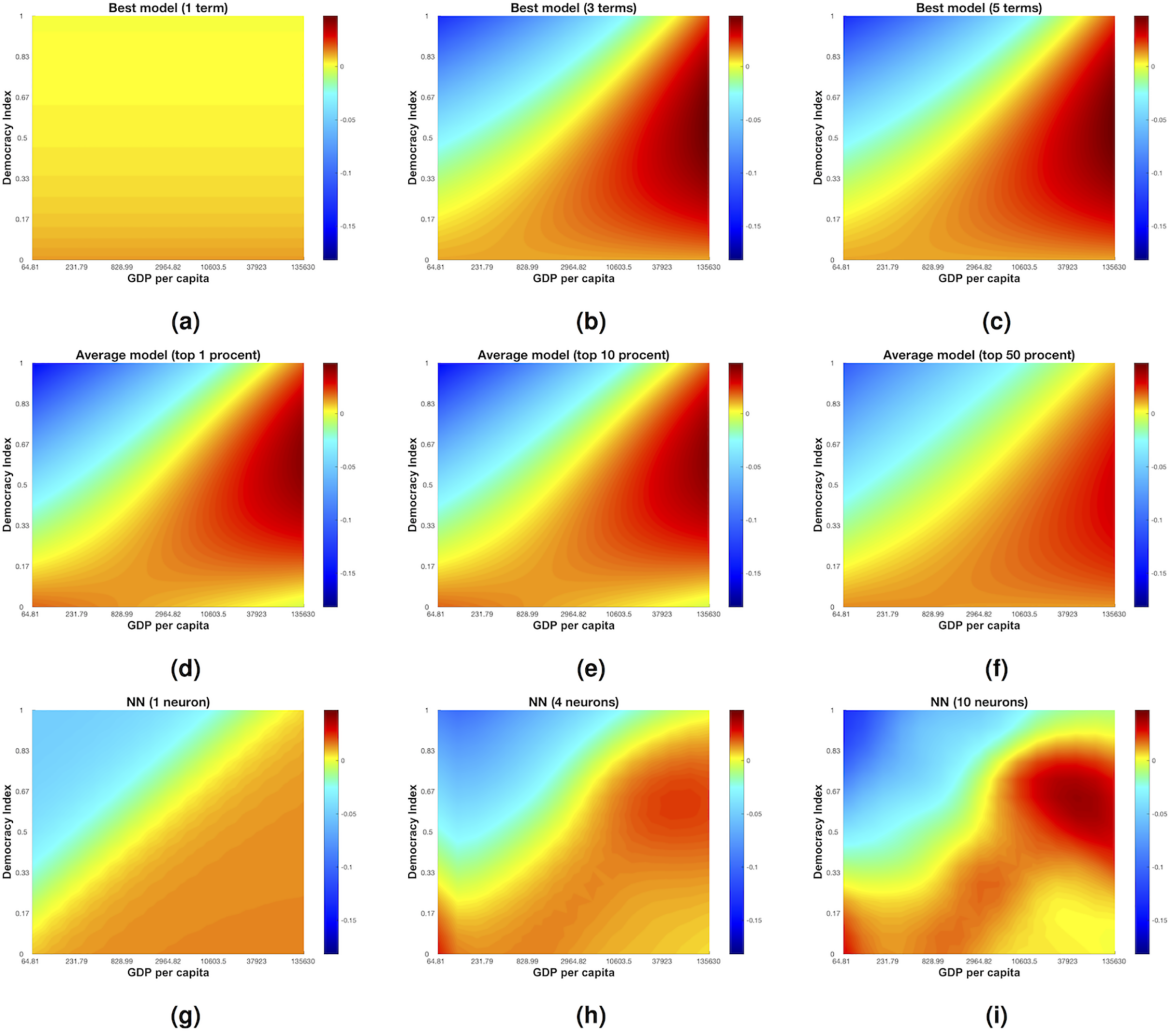
Change in democracy (D). The three top figures in black (Fig 3 a,b,c) are visualizations of the changes in democracy for best models with one (Fig 3 a), three (Fig 3 b) and five (Fig 3 c) terms. The three figures in the vertical middle (Fig 3 d,e,f) represents 1% (Fig 3 d), 10% (Fig 3 e) and 50% model averaging models. The three figures at the bottom is representations of feedforward neural networks with 1 (Fig 3 g), 4 (Fig 3 h) and 10 (Fig 3 i) neurons in the hidden layer.

We can visualize our models using two dimensional heat maps. In Fig 3(a)–3(c) we plot the best one-, three- and five-terms models respectively for democracy. A visual comparison of the three- (Eq 19) and five-terms models shows that the extra complexity of the latter does not significantly change the predicted dynamics. The average models (shown in Fig 3(d)–3(f)) show a similar dynamics to the Bayesian best model. The non-parametric neural network models in Fig 3(g)–3(i) also show a similar behavior. The consistency of the pattern found in the change in democracy using these three different approaches suggests that, even though the *R*^2^ are relatively small, these models reflect a genuine relationship between democracy and GDP over the past 30 years.

The Bayesian best model for log GDP per capita (Eq 20) has a *R*^2^ of 0.7%, which is significantly lower then for our best model for democracy. Similarly, the best average model (10%) has an *R*^2^ of 1%. The best neural network model (six neurons) has an *R*^2^ of 1.8%, which is twice as large as the one found for the Bayesian best model. Such big difference, combined with an extremely low *R*^2^, casts serious doubt on the reliability of the log GDP per capita model. Moreover, the best one-term model for log GDP per capita is the 1.1% constant change model and is ranked 360 out of 9401 models. This high rank of the constant change model tells us that even the simplest model, not including democracy nor GDP per capita, is deemed to be almost as good as our ‘best model’, thereby weakening our belief in our model of GDP per capita.

**Fig 4:**
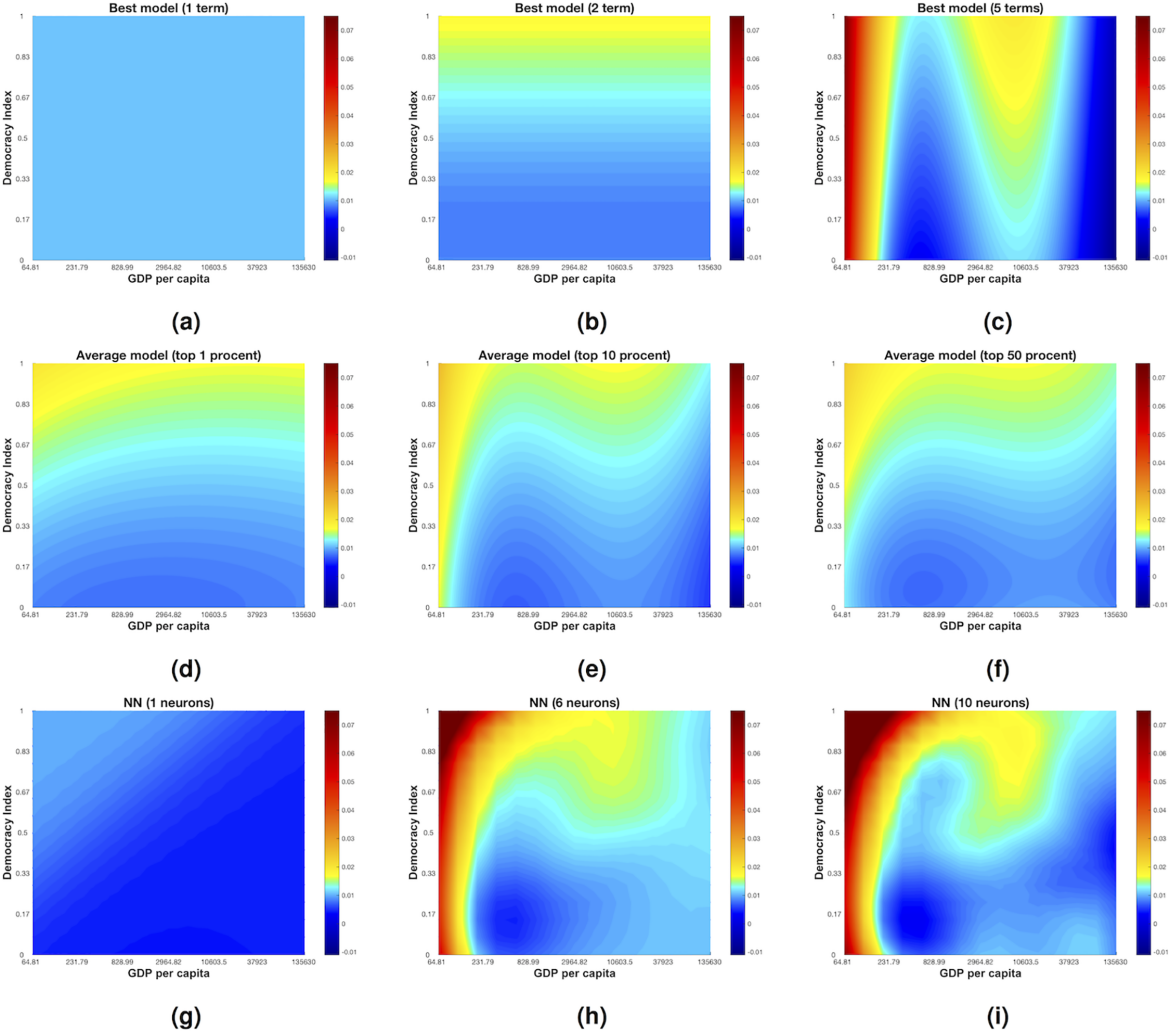
Change in log GDP per capita (*G*). The three top figures (Fig 4 a,b,c) are visualizations of the changes in *G* per capita for best models with one (Fig 4 a), two (Fig 4 b) and five (Fig 4 c) terms. The three figures in the vertical middle (Fig 4 d,e,f) represents 1% (Fig 4 d), 10% (Fig 4 e) and 50% model averaging models. The three figures at the bottom is representations of feedforward neural networks with 1 (Fig 4 g), 6 (Fig 4 h) and 10 (Fig 4 i) neurons in the hidden layer.

A visual comparison of the Bayesian best model for log GDP (Fig 4b) with models with less and more terms (Fig 4a and 4c), with the average models (Fig 4(d)–4(f)), and with neural network models (Fig 4(g)–4(i)) reveals significant differences between models found with different approaches. The average models are similar to the Bayesian best model, featuring a slightly more complex dynamics. The best (6 neurons) neural network model shows similarities with the 5-term Bayesian best model, but not with the highest-ranked two-terms model. Taken together, these results question the validity and reliability of Eq (20) as a model for the change of GDP per capita.

Finally, we test the robustness of our Bayesian best models by comparing all 9401 possible one- to five-term configurations. We argue that terms that appear repeatedly in different highly-ranked models are more likely to be a robust description of the data. In Table 2 we report the eight most frequent terms among the top ranked 100 model configurations for both democracy and log GDP per capita. The frequency of two-terms combinations are also presented, showing how likely it is for two particular terms to appear together. If two terms appear together frequently then we infer that this combination of terms is more robust.

**Table 2:** Robustness of terms for democracy (*D*) and log GDP per capita (*G*). The three columns furthest to the left shows the most eight most frequently recurring terms among the top 100 models for (*D*) and (*G*). The columns to the right of show how often the terms appear in combination to each other. Red bars means a positive sign on the term and blue bars negative.

**Democracy**									
**Frequency per term**			**Frequency of combination**						
**Term model**	Procent		−*D*^2^	−*D*^3^	$\frac{-D}{\left(1+G\right)}$	−*D*	$\frac{1}{{\left(1+D\right)}^{3}}$	*D*	$\frac{1}{{\left(1+D\right)}^{2}}$
*DG*	0.82		0.41	0.41	0.30	0.25	0.17	0.18	0.00
−*D*^2^	0.50			0.00	0.30	0.17	0.07	0.09	0.08
−*D*^3^	0.49				0.17	0.08	0.12	0.05	0.08
−*D*/(1 + *G*)	0.48					0.00	0.07	0.09	0.17
−*D*	0.25						0.04	0.06	0.00
1/(1 + *D*)^3^	0.19							0.04	0.02
*D*	0.19								0.01
1/(1 + *D*)^2^	0.17								
**log GDP per capita**									
**Frequency per term**			**Frequency of combination**						
**Term model**	Procent		*D*	$\frac{1}{{\left(1+D\right)}^{3}}$	$\frac{G}{\left(1+D\right)}$	*D*^2^	$\frac{1}{{\left(1+D\right)}^{2}}$	$\frac{1}{{\left(1+G\right)}^{3}}$	$\frac{1}{\left(1+D\right)}$
*D*/(1 + *G*)	0.40		0.00	0.11	0.08	0.01	0.08	0.03	0.04
*D*	0.32			0.10	0.06	0.00	0.06	0.06	0.03
1/(1 + *D*)^3^	0.23				0.02	0.03	0.00	0.01	0.00
*G*/(1 + *D*)	0.16					0.02	0.02	0.03	0.01
*D*^2^	0.16						0.02	0.03	0.02
1/(1 + *D*)^2^	0.16							0.01	0.00
1/(1 + *G*)^3^	0.13								0.00
1/(1 + *D*)	0.11								

For democracy, the terms *DG* and −*D*^2^, appear in both the best two-term and three-term models, and are the two most frequent terms among the 100 configurations with highest Bayes factor. We use 100 configuration to test if the terms are robust for the ∼1% of tested models to see if the terms in the best models are present still when we look beyond only the best models. The term *DG* is involved in 82% of these models. Half of these models include the term −*D*^2^, while the other half include the term −*D*^3^. These two self-limiting terms, −*D*^2^ and −*D*^3^, never appear together in the same model and clearly play the same role in fitting the data. This recurrence supports our belief that the democracy model extracted within our approach captures a genuine aspect of the relationship between democracy and GDP.

The third term in Eq (19), 1/(1 + *D*)^3^, does not appear as frequently and does not have as big impact on the change in democracy as the other two terms. This seems to suggest that the most robust description of the relationship between the rate of change of democracy and GDP is

$$\begin{array}{ccc} \frac{dD}{dt} & ∼ & D(0.18G-0.15D). \end{array}$$ (21)

Although this model differs from the best model in Eq (19), this functional form combines highest *R*^2^ value, highest model ranking, robust combination of terms, and highest interpretability, which makes it the most explanatory and robust model for democracy.

For log GDP per capita (Eq 20), the terms *D* and 1/(1 + *D*)^3^ are found in only the 32% and 23% of the 100 top-ranked configurations. The most frequent term, *D*/(1 + *G*), is found in 40% of the top 100 configurations. There are few consistent pairings of terms among the top 100 models, i.e. *D* together with 1/(1 + *D*)^3^ (10%) and *D*/(1 + *G*) with 1/(1 + *D*)^3^ (11%), while the other combinations are evenly distributed. This seems to further indicate that the best model for log GDP per capita is not reliable in describing the available data.

Our chosen best models for democracy and log GDP per capita are stable with respect to reasonable changes in the g-prior’s parameter *g*. In particular, our approach returns the same ‘best models’ for both *D* (Eq 19) and *G* (Eq 20), which are found at *g* = 3445, but with changed configurations’ ranking. For example, by doubling *g* (*g* = 2 × 3445 = 7890) complicated models get punished more harshly and are thereby ranked lower. Halving *g* (*g* = 3445/2 = 1722.5) also gives us the same ‘best models’, but complicated models are less punished. Instead, we obtain significantly different models when the parameter *g* gets very large (*g* = 10^100^) or very small (*g* < 300). In these extreme cases, the Bayesian selection favors respectively the one-term model presented in Table 1, and models with many terms, even though these terms are not consistent with our best one- to five-term models in Table 1.

### 3.3 Surrogate data testing

Even the best model for the changes in democracy (Eq 19) explains only a small part of the dynamics. A way to further investigate how robustly we can detect such a weak signal in noisy data is using surrogate data. We generated surrogate data using Eq (21) for the changes in democracy and Eq (20) for changes in log GDP per capita. The surrogate data is generated with the same number of initial countries and time steps as in the original data and all other parameters are chosen to be consistent with the methodology presented in section 2.2. We sampled the initial values for the surrogate data set from the initial values in the original data. We use noise terms derived directly from empirical data ($\sigma_{D}^{2}=0.08$, $\sigma_{G}^{2}=0.02$).

Even though we used a two term model (Eq 21) to generate data for democracy to fit the model we found that the following four term model, from the first fitting of surrogate data, was a typical best model for democracy,

$$\begin{array}{c} \frac{dD}{dt}=0.186DG-0.154D^{2}+\frac{0.208}{{\left(1+D\right)}^{3}}-\frac{0.181}{{\left(1+D\right)}^{2}}. \end{array}$$ (22)

The fact that the resulting model is very similar, albeit with extra terms, provides additional evidence that our method is robust in the presence of noise. There were, however, additional spurious terms in the best models which may help us better understand our results. In particular, it is interesting to note, that the term 1/(1 + *D*)^3^ in Eq (22) also arose in the overall best model (Eq 19). This strengthens our belief that our final model (i.e. Eq 21) for the dynamics in democracy is more parsimonious than a model including 1/(1 + *D*)^3^. It is plausible that these two latter terms is simply an artifact arising from our choice of prior i.e., the parameter *g* is set to small (see the discussion in the end of section 3.2), rather than a genuine statistical relationship.

We also performed inference on the 1000 best model found for changes in democracy, using 1000 different sets of surrogate data. We found that the most frequent best model (609 out of 1000) was,

$$\begin{array}{c} \frac{dD}{dt}∼DG-D^{3}+\frac{1}{{\left(1+D\right)}^{3}}-\frac{1}{{\left(1+D\right)}^{2}}, \end{array}$$ (23)

Note that we get the same model as in Eq (22), with the difference that *D*^2^ is exchanged with *D*^3^. Table 3 shows the frequency of occurrence of the different terms, and how often these terms are found in the same model configuration within the top 100 models, for all 1000 surrogate data sets, for both democracy and log GDP per capita. For democracy, the best explicit models and the most frequent top configurations were found to be robust—with just small differences in coefficient values. The fact that the terms *D*^2^ is exchanged with *D*^3^—note that *D*^2^ and *D*^3^ is very similar when *D* ∈ [0, 1], with *D*^3^ is picked more frequently then *D*^2^ together with *DG* in models with more terms— indicates that the terms should be interpreted in qualitative terms, rather than in terms of their specific exponents.

**Table 3:** Robustness of terms (surrogate data) for democracy (*D*) and log GDP per capita (*G*). The three columns furthest to the left shows the most eight most frequently recurring terms among the top 100 models for (*D*) and (*G*) for 1000 generated surrogate data sets. The columns to the right of show how often the terms appear in combination to each other. Red bars means a positive sign on the term and blue bars negative.

**Democracy**							
**Frequency per term**			**Frequency of combination**				
**Term in model**	Procent		*DG*	1/(1 + *D*)^3^	−*D*/(1 + *G*)	*D*	−1/(1 + *D*)
−*D*^3^	0.75		0.50	0.41	0.23	0.19	0.19
*DG*	0.62			0.39	0.05	0.11	0.19
1/(1 + *D*)^3^	0.56				0.15	0.11	0.16
−*D*/(1 + *G*)	0.35					0.14	0.08
*D*	0.30						0.07
−1/(1 + *D*)	0.28						
**Log GDP per capita**							
**Frequency per term**			**Frequency of combination**				
**Term in model**	Procent		*D*^2^	*D*/(1 + *G*)	*Const*	1/(1 + *D*)^3^	1/(1 + *D*)^2^
*D*	0.29		0.01	0.01	0.02	0.08	0.07
*D*^2^	0.27			0.02	0.06	0.02	0.03
*D*/(1 + *G*)	0.24				0.01	0.07	0.05
*Const*	0.22					0.02	0.01
1/(1 + *D*)^3^	0.20						0.00
1/(1 + *D*)^2^	0.18						

For log GDP per capita, the best models changed a lot for every realization. The term *D* and 1/(1 + *D*)^3^ shows up as the most frequent and the fifth most frequent terms in Table 3. However, the distribution of the top terms is very flat, indicating that the terms show up relatively equally among the best models. This further supports our previous conclusion that change in GDP can not be reliably modeled by democracy.

## 4 Discussion

In this paper, we accomplish two main goals. First, we improve upon the approach proposed in [15] by fitting data to equation-based ‘best models’ through Bayesian linear regression. Second, we develop a way of testing the robustness of the obtained models by comparing our method with two prediction-oriented methods: model averaging and neural networks. We discuss these two points in turn.

The strength of the approach developed in [15] is that it provides relationships between the variables, log GDP and Democracy in this case. They chose a two-term model (Eq 1) as the best model for democracy. Interestingly, their model displays the same threshold behavior as our best model for democracy (Eq 19). Our Fig 3 has clear similarities with the heat map of change in democracy presented in Fig 3a in [15] for all three modelling methods used in our paper. So even though we find different explicit expressions for the change in democracy, the overall dynamics is similar. The only visual difference is that our model gives a higher value for democracy, where the change is zero, for the low GDP per capita region. However, the terms selected are not the same as in our model. The primary reason for this difference is because we use more data: 174 countries instead of 74, and rescaled the indicator variables. Moreover, although being a convenient way of fitting equation-based ‘best models’ to data, their use of uniform flat priors makes analytical calculations not attainable for the posterior distributions. For this reason, they had to turn to numerical estimations, which caused loss of information and prevented them from studying and comparing all possible models, to check the robustness of the terms chosen. Furthermore, we argue that our final expression Eq (21) is not only checked for robustness (Table 2), it is also easier to interpret than the final expression in [15]. For these reasons, we argue that our model is a better description of the dynamics of democracy and log GDP per capita.

In our approach, we use Bayesian linear regression and a mathematically convenient prior [46, 48]. This choice allows us to get closed form expressions for the marginal likelihoods and to significantly lower the computational burden. As a result, we can quickly compare and rank all model configurations, and study the frequency of single and combined terms in different models, thus performing an accurate analysis of the robustness of our ‘best model’.

**Fig 5:**
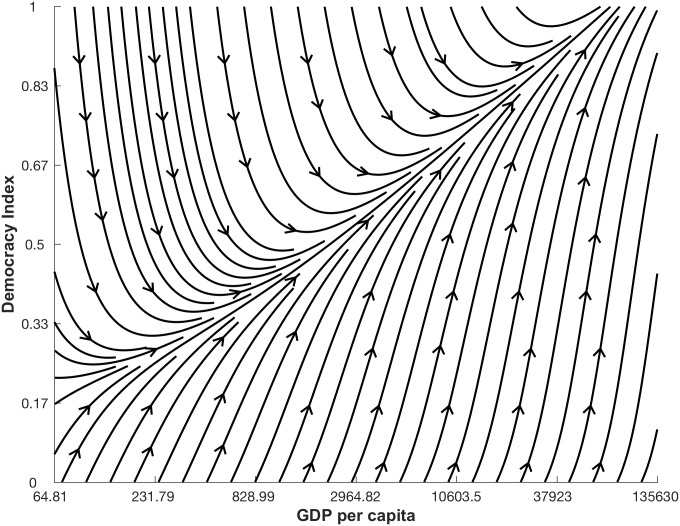
Relation between democracy and GDP per capita. Dynamics of the relation between democracy (*D*) and (*G*), in USD, using best models, Eq (19) (*D*) and Eq (20) (*G*), displayed using linear interpolated streamline plot.

Social systems often display nonlinear interactions between indicator variables [8–10], making their study an interesting challenge. For example, Fig 1 shows a clearly nonlinear relation between democracy (*D*) and log GDP per capita (*G*). Here, we had the general goal of modelling this relation by distinguishing genuine interactions from noise. Fig 5 shows the best relationship between democracy and log GDP per capita we can extract by applying our methodology. According to this plot, and thus our methodology, once noise is filtered out *D* and *G* are connected by a simple threshold relationship. Moreover, we can extend our estimates of the dynamics in areas of the state space (*D*, *G*) where we have no data measured.

Our method relies on us comparing our explanation-oriented model with more predictive-oriented alternatives such as artificial neural networks. Artificial neural networks can be seen as universal estimators [22] and are widely used to study nonlinear systems appearing in social-economical systems [28]. Here we used ANNs as a benchmark to assess if the tradeoff between interpretability and predictive power is satisfactory. In our example of democracy and GDP per capita we found our modelling approach to give satisfactory results for democracy, but we also concluded the best model for GDP per capita was insufficient. We also used a Bayesian model-averaging approach as benchmark to account for model uncertainties in our ‘best models’.

In the social sciences, it is common that models have low statistical power, because of their inherent complexity of the system and high levels of noise. Both the model for democracy and the model for log GDP have low *R*^2^. In the case of changes in democracy, by subjecting the original equation-based model to a sequence of comparisons—first to model averages, second to neural networks and finally surrogate data, from the equation itself—we are able to increase our confidence in the model as a description of the underlying system dynamics. We find that the exponents used for modelling the data, i.e. comparing *D*^2^ or *D*^3^, can be exchanged, but the overall negative and positive feedbacks captured by (Eq 19) are a robust feature of the data. Despite the low *R*^2^ of around 4% we have captured the underlying relationships. In contrast, when we subjected the GDP model (which has *R*^2^ of around 1%) to the same battery of tests, it repeatedly failed to give robust results. The techniques we have presented here, thus provide a way of interrogating and increasing our confidence in a model, even when it provides very weak explanatory power in a statistical sense.

A question that always arises in study like the one about the choice of priors. We choose to mimic a non-informative prior by setting the shape and rate parameters to be very small. This choice is common, and used for example in [63], but we have to be careful when using these choices when performing inference, since it can be sensitive to the small values, as implied in [64]. In our application, we can not see any problems regarding this, but users of our methodology should be aware of these potential complications.

Having benchmarks of neural networks and model averages to compare an equation-based model with is especially important if we wish to move up in dimensionality i.e. when studying multivariate coupling functions arising when studying systems with more than two indicator variables [65, 66]. Adding variables into our social system makes them harder to visualize using two-dimensional heat maps, as we did in Figs 3 and 4. With three-variables models we could visualize the relations between indicators and compare models using three-dimensional plots, but if we want to go even further up in dimensionality [3, 42] we might need to assume that some variables are held constant—assuming that there are interactional terms to these additional variables. This would make the global relations harder to study. Providing explicit equation-based models and a way to test their robustness, our Bayesian-based approach is a valuable tool for understanding the relationships underlying complex social systems.
